# Deformable Self-Propelled Micro-Object Comprising Underwater Oil Droplets

**DOI:** 10.1038/srep31292

**Published:** 2016-08-09

**Authors:** Taisuke Banno, Arisa Asami, Naoko Ueno, Hiroyuki Kitahata, Yuki Koyano, Kouichi Asakura, Taro Toyota

**Affiliations:** 1 Department of Applied Chemistry, Faculty of Science and Technology, Keio University, 3-14-1 Hiyoshi, Kohoku-ku, Yokohama 223-8522, Japan; 2 Department of Basic Science, Graduate School of Arts and Sciences, The University of Tokyo, 3-8-1 Komaba, Meguro-ku, Tokyo 153-8902, Japan; 3 Department of Physics, Graduate School of Science, Chiba University, 1-33 Yayoi-cho, Inage-ku, Chiba 263-8522, Japan; 4 Research Center for Complex Systems Biology, The University of Tokyo, 3-8-1 Komaba, Meguro-ku, Tokyo 153-8902, Japan

## Abstract

The self-propelled motion with deformation of micrometer-sized soft matter in water has potential application not only for underwater carriers or probes in very narrow spaces but also for understanding cell locomotion in terms of non-equilibrium physics. As far as we know, there have been no reports about micrometer-sized self-propelled soft matter mimicking amoeboid motion underwater. Here, we report an artificial molecular system of underwater oil droplets exhibiting self-propelled motion with deformation as an initial experimental model. We describe the heterogeneity in a deformable self-propelled oil droplet system in aqueous and oil phases and at their interface based on the behavior and interaction of surfactant and oil molecules. The current results have great importance for scientific frontiers such as developing deformable micro-swimmers and exploring the emergence of self-locomotion of oil droplet-type protocells.

Micrometer-sized self-propelled objects in water, such as micrometer-sized robots^1^, gene-engineered cells^2^, and so on^3^, have drawn much attention because of their potential application as underwater carriers and/or probes for exploring and curing environmental or biological systems. Living mobile cells can respond to circumstances and move using far less energy by machines or robots of comparable size. However, the fact that they are biological limits their application. Therefore, the construction of chemically organized soft systems in which molecular assemblies such as polymer networks exhibit the ability to mimic biological/cellular locomotion has gradually captured the attention of researchers and investors^4^,^5^,^6^,^7^. Recently, micrometer-sized emulsion droplets^8^ and giant vesicles^9^,^10^,^11^,^12^ in water exhibiting locomotion have been reported. Moreover, the chemotactic motion of droplets under external stimuli such as gradient fields of chemical compounds^13^,^14^,^15^,^16^ and light irradiation^17^,^18^ has been demonstrated. These self-propelled objects composed of soft matter have provided an important and emerging topic in non-equilibrium physics. In such studies, Ohta and Ohkuma provided a generic theory on the relationship between locomotion and shape change based on the symmetry-breaking^19^. The comparison between such generic analyses and experimental results is important, especially on the microscopic scale, for understanding cell locomotion, and there are few microscopic systems that overcome interfacial tension and consequently exhibit deformation.

As far as we know, there have been no reports on micrometer-sized self-propelled soft matter mimicking amoeboid motion underwater. Like white blood cells, *Dictyostelium discoideum*, and so on^20^, amoeboid motion is a type of cell locomotion associated with fluctuations in body shape. Hence, micrometer-sized self-propelled soft matter exhibiting such deformation could be applied as underwater carriers or probes in very narrow spaces, similar to white blood cells invading blood vessels to attack tumors beneath the vessel. Herein, we report the development of an artificial molecular system of underwater oil droplets exhibiting self-propelled motion with deformation within a submillimeter-sized compartment as an initial experimental model. This oil droplet system is based on oil droplet locomotion driven by the Marangoni effect and the transport of molecules around the oil droplet surface in a surfactant solution^8^. In this study, we focused on environmentally benign compounds having alkyl chains, which can be synthesized by hydrothermal reactions under conditions similar to those at the field of origin of life^21^,^22^,^23^,^24^. Such compounds accumulate in submillimeter-sized compartments of chimneys at hydrothermal vents^25^,^26^. Moreover, referring amoeba cells exhibiting sol‒gel transition of the cytosol during their locomotion^27^, our oil droplet system includes a mixture of two miscible compounds; a fatty aldehyde (1-undecanal, C10A) and an alcohol (1-decanol, C10OH). The attraction between C10A and C10OH induced spatial heterogeneity in the mixed oil droplets. These compounds also formed acetal (diC10A) under acidic conditions (Fig. 1a). Therefore, when these oil droplets were dispersed in an acidic aqueous solution of the surfactant *n*-hexadecyltrimethylammonium bromide (C16TAB), this heterogeneity resulted in differences in the adsorption and/or dissolution of the surfactant molecules at the surface and core of the oil droplets. The deformable self-propelled oil droplets are thus realized in this system (Fig. 1b).

## Results

### Dynamics of oil droplets composed of fatty aldehyde and alcohol

**Figure 2:**
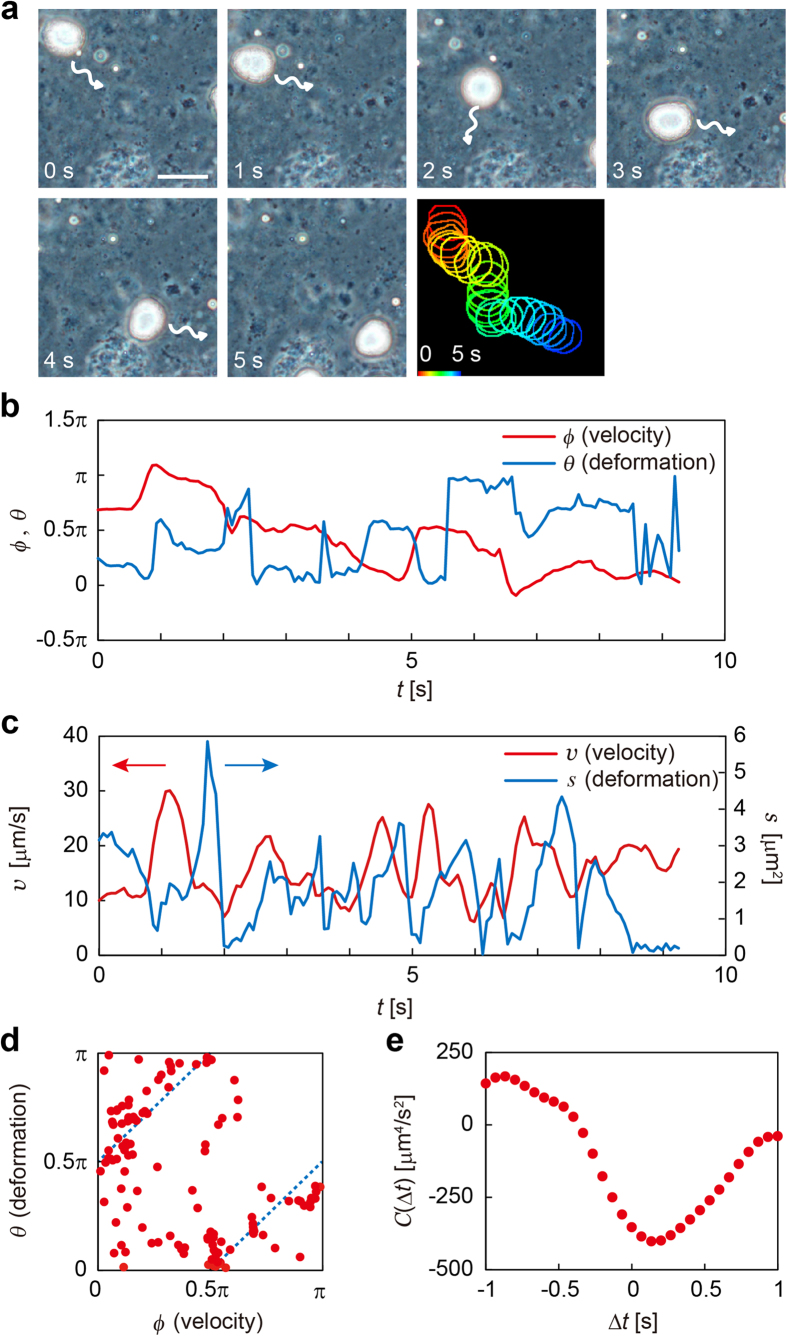
Microscopy image analysis of deformable self-propelled oil droplets. (**a**) Typical sequential micrographs (time interval = 1 s) of deformation of self-propelled oil droplets in the dispersion of C10A/C10OH (60/40 mol%) prepared in an acidic solution (pH 2) of C16TAB (50 mM) 2 min after preparation of the emulsion. The arrows represent the direction of travel of the self-propelled oil droplet. Scale bar: 30 μm. The superposed merged image of the rims of the deformed self-propelled oil droplets (the color was digitally edited) is also shown. (**b**) Time series of the direction of motion, *ϕ* (red), and that of the major-axis direction of the elliptic deformation, *θ* (blue). (**c**) Time series of the velocity, *v* (red), and the amplitude of the elliptic deformation, *s* (blue). (**d**) Plots of *θ* against *ϕ*. Blue dotted lines correspond to *θ* = *ϕ* ± π/2. Because plot points are distributed along the blue dotted lines, it is suggested that the droplet moves in the direction of its minor-axis. (**e**) Plot of *C*(Δ*t*) defined in Eq. (2). The negative values around Δ*t* ≈ 0 s mean that the motion is in the direction of minor-axis. The correlation is strongest at Δ*t* ≈ 0.1 s, which means the deformation follows the motion of the droplet with a delay time of approximately 0.1 s.

First, we investigated the effect of oil composition on oil droplet dynamics in an emulsion system (200 μL) containing C16TAB (50 mM) and 0.01 M HCl at room temperature (23–25 °C). C10A and C10OH were mixed beforehand and dispersed into the surfactant solution. The emulsion samples were packed into a 280 μm-thick compartment consisting of two slide glasses and a spacer. The oil droplets therein were then observed under a phase contrast microscope. When the molar ratio of C10A/C10OH was 60/40 mol%, we observed self-propelled motion of the oil droplets, the shapes of which changed autonomously (see Supplementary Movie 1). The frequency of the shape change was in the range of 1–10 s^−1^ (Fig. 2a). To evaluate the change in shape of the oil droplets as seen in the microscopy images, we fitted the cross section of an oil droplet to an ellipse and measured the amplitude, *s*, and the direction of the major axis, * θ*, in the elliptic deformation. A detailed explanation of the fitting and calculation of *s* and *θ* can be found in the Supplementary Information (see Supplementary Fig. 1). We also measured the center of mass of the droplet and calculated its velocity.


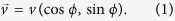


The time series of the direction of velocity, *ϕ*, and of the major axis direction of elliptic deformation, *θ*, are shown in Fig. 2b. Those of the absolute value of velocity, *v*, and the amplitude of the elliptic deformation, *s*, are shown in Fig. 2c. As shown in Fig. 2d, the difference between *ϕ* and *θ* was distributed around π/2. In order to confirm the correlation, we plotted *θ* versus *ϕ* for each time (Fig. 2d). The resulting plot was distributed along two lines, *ϕ* = *θ* ± π/2, which suggests that the droplet tended to move in the direction of its minor-axis. In Fig. 2c, the peak of *s* seems to follow that of *v*, which suggests a time correlation between *s* and *v*. Thus, we calculated the time correlation between *s* and *v* in the form of


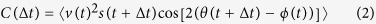


The results are shown in Fig. 2e. The plot has a local minimum at Δ*t* ≈ 0.1 s, which suggests that there is a delay of approximately 0.1 s between the deformation change and the velocity change. By this geometric analysis, we deduced that the self-propelled oil droplet had a tendency to become slightly squashed perpendicular to its direction of motion and that its direction changed frequently.

Not only the time correlation analysis of deformation and velocity but also the analysis of flow around the oil droplets gives us crucial clues into the driving force of the self-propelled motion and deformation. We traced water-dispersed fluorescent microspheres (diameter = 1 μm) in the C16TAB solution (containing 0.1 M HCl), where the C10A/C10OH oil droplets (60/40 mol%) exhibited self-propelled motion and deformation, under a fluorescence microscope (see Supplementary Fig. 2 and Movie 2). The fluorescent microspheres at the back of the oil droplet flowed in the direction opposite that of the oil droplets about 3 times faster than those at the front of the oil droplets. This implies that the back of the self-propelled oil droplets forced the surrounding water advection outward, resulting in the deformation of the oil droplets.

### Effects of molecular structure of components on droplet dynamics

Because advection in the vicinity of the oil/water interface is caused by surfactant adsorption, to evaluate the effect of the surfactant on the deformation, we observed oil droplet dynamics in 0.01 M HCl containing C16TAB in the concentration range of 5‒50 mM (Supplementary Table 1). A more concentrated surfactant solution tended to facilitate self-propulsion and changes in the shapes of the droplets. In 5 mM C16TAB, no significant motion was observed (entry 1 in Supplementary Table 1). In the range of 10‒30 mM C16TAB, the deformable self-propelled oil droplets were observed for a few minutes from the start of observation (entries 2‒4 in Supplementary Table 1). On the other hand, in the presence of 40 mM and 50 mM C16TAB, self-propelled motion with deformation of the oil droplets sustained for 26 and more than 60 min, respectively (entries 5 and 6 in Supplementary Table 1). These results clearly indicate that a relatively large amount of C16TAB was essential for the sustaining the deformation of the oil droplets.

**Figure 3:**
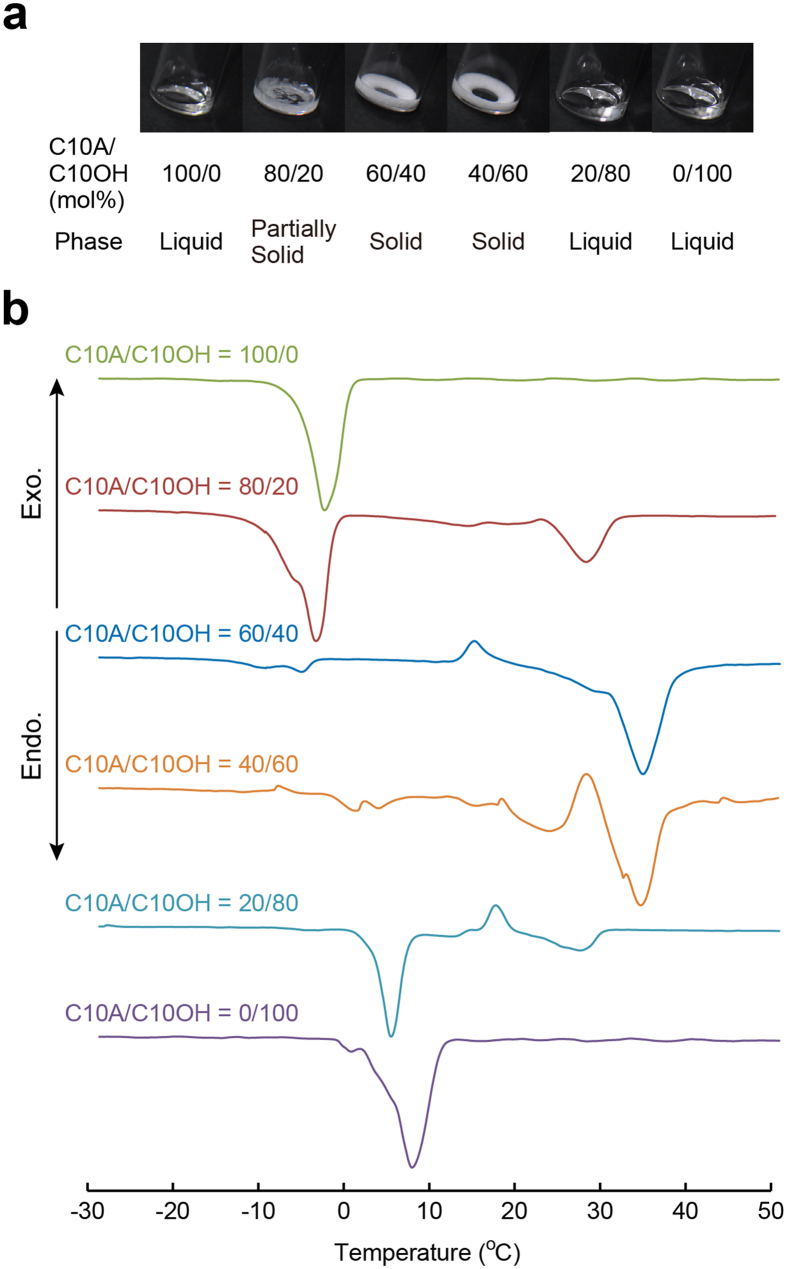
Thermal properties of oils. Macroscopic observations and thermal properties of oil components prepared by mixing C10A and C10OH. The (**a**) photographs and (**b**) 2^nd^ heating DSC curves of the individual and mixed oils.

These unique dynamics were also strongly influenced by the molar ratio of C10A and C10OH (Supplementary Table 2). Deformation was observed when we used the oil mixture with molar ratio of C10A/C10OH from 60/40 to 20/80. Note that mixed oil components composed of C10A/C10OH with molar ratios of 80/20, 60/40, and 40/60 transitioned to a solid phase 5 min after the preparation of each mixed oil component at room temperature (22 °C) (Fig. 3a). To investigate the thermal properties of C10A, C10OH, and mixtures of the two, we carried out differential scanning calorimetry (DSC) measurement (Fig. 3b). A single endothermic peak appeared in C10A and C10OH and their melting points (*T*_m_) were −3.6 and 6.8 °C, respectively. On the other hand, the mixtures exhibited higher *T*_m_ values than the individual oils, and they had some exothermic and endothermic peaks, suggesting that some complex phases were formed in the mixtures. This is probably owing to the strong attractive forces between C10A and C10OH, such as hydrogen bonding and dispersion force.

Next, to investigate the dependency of the oil and surfactant molecules on the droplet locomotion mode, we prepared emulsion samples using various cationic surfactants and oil components and observed the droplet dynamics. We did not observe deformed self-propelled oil droplets composed of C10A and other oil molecules such as decane and nonanoic acid. Their intermolecular interactions with C10A are different from C10OH. Also, in a 50 mM solution of C12TAB, deformed self-propelled oil droplets were not observed. These results suggest that the combination of oil and surfactant molecules, which induces the time-dependent spatial heterogeneity in the droplets, was essential for self-propelled motion with deformation.

### Deformable self-propelled oil droplets in various dispersions

We also focused on acetal formation inside the combined C10A/C10OH oil droplets because the acetal diC10A produced by the reaction between C10A and C10OH became the fourth component of the self-propelled oil droplets. We previously reported that acetalization between a benzaldehyde and an alcohol occurs by the dissolution of acids (H_3_O^+^) in the self-propelled oil droplet in the presence of C16TAB^28^. We thus investigated the relationship between oil droplet dynamics and the pH and ionic strength of the dispersion, and we conducted further reference experiments (Supplementary Table 3). Using HCl concentrations of 0, 0.001, 0.01, and 0.1 M, deformed self-propelled droplets were observed at 0.01 M and 0.1 M HCl. For HCl concentrations below 0.01 M, the self-propelled droplets did not exhibit deformation. We thus investigated whether the dynamics of the oil droplets involved a chemical reaction between C10A (0.044 mmol) and C10OH (0.029 mmol) for 60 min. The^1^ H NMR spectra of the lyophilized residue of a dispersion containing 0–0.1 M HCl in the presence of C16TAB (50 mM) were analyzed to determine the production of diC10A (Supplementary Fig. 3). Under these conditions, some amounts of diC10A were produced (Supplementary Table 3). It is noted that, when we synthesized diC10A and dispersed it in a 50 mM C16TAB solution with 0.01 M HCl, none of the diC10A oil droplets exhibited any motion, even 30 min after specimen preparation. Using^1^ H NMR, we found that 60% of diC10A was hydrolyzed within 10 min and that the hydrolysed amount was almost constant thereafter in this dispersion. Moreover, we observed the dynamics of oil droplets composed of C10A, C10OH, and 1–10 mol% diC10A (relative to the C10A/C10OH oil components) in the same acidic solution containing C16TAB (Table S4). The motion time of the oil droplets decreased with increasing diC10A amount, and no deformation was observed when more than 3 mol% diC10A was added to the oil components. This clearly indicates that diC10A was produced in the oil droplets exhibiting deformation; however, as the diC10A concentration increased, it behaved as an inhibitor for droplet motion in the system.

Finally, to clarify the role of hydrochloride, we investigated the effect of electrolytes and their concentration on oil droplet dynamics (Supplementary Table 5). Deformed self-propelled oil droplets were observed in tested solutions of the monovalent and divalent electrolytes NaCl (0.001–0.1 M), NaBr (0.01 M), and MgCl_2_ (0.01 M). Because oil droplets exhibited only self-propelled motion in the absence of electrolytes (entry 6 in Supplementary Table 5), it is implied that the electrolyte was needed for inducing deformation.

## Discussion

In this study, we constructed micrometer-sized self-propelled oil droplets exhibiting deformation in a submillimeter-sized compartment. This novel machinery was derived in an emulsion system that included the mixed oil droplets composed of 60/40 mol% C10A/C10OH and an acidic surfactant solution of C16TAB. The mixed oil itself formed a solid phase at the observation temperature. The production of diC10A was involved in the deformation, but the motion was suppressed as diC10A accumulated in the droplets. The presence of an electrolyte at concentrations above 0.001 M in the emulsion was necessary for deformation. The rate of adsorption of ionic surfactant molecules onto the interface increased by the addition of electrolytes, which reduced the charge repulsion between surfactant molecules^29^. Also, surfactant molecules were easily absorbed into the oil phase in terms of distribution equilibrium. We thus assume that the deformation of the self-propelled droplets in this system is associated with the dynamic behavior of the surfactant molecules, such as adsorption onto the oil‒water interface and absorption into the core of the oil droplets.

Upon this assumption, we now discuss the mechanism of deformation of the self-propelled oil droplets. There are three possible candidates for the mechanism of the deformation. The first one is the hydrodynamical effect, in which the droplet motion induces a pressure change inside the droplet, resulting in the deformation. The second one is the imbalance of Laplace pressure, in which the surface-active materials are transported by diffusion. The subsequent change in the interfacial tension induces the deformation by disrupting the balance of the Laplace pressure. The third candidate is also a hydrodynamical effect; however, it does not originate from the motion of the droplet but rather from the convective flow induced by the Marangoni effect. An evaluation of these three candidates is based on the observed time delay of approximately 0.1 s between motion and deformation. The characteristic time necessary to propagate the pressure difference across a droplet diameter, *d*, of 20 μm was calculated to be 0.4 ms, which is much smaller than 0.1 s. Here, the characteristic time was obtained from the kinetic viscosity *κ* = 10^−6^ m^2^ s^−1^, and the relation between the viscosity constant and the characteristic time and scale, i.e., *d*^2^ ≈ *κτ*, where *τ* is the characteristic time. Thus, the hydrodynamic interaction through viscosity would not result in the time delay observed in this measurement, and thus the first candidate, the hydrothermal effect, is not the mechanism of deformation. Then, we consider transport by diffusion. The diffusion coefficient, *D*, is approximately equal to 10^−9^ m^2^ s^−1^, and its relationship to diffusional transport is *Dτ* ≈ *d*^2^, where *τ* is calculated to be approximately 0.1 s. This characteristic time is comparable to the obtained data, and thus diffusional transport may play a significant role in the correlation between motion and deformation. The third candidate is transport by convective flow. The velocity of the droplet, *v*, was observed to be approximately 10 μm s^−1^, and the convective flow inside and outside the droplet are of the same order as the droplet velocity, *v*. The characteristic time, *τ*, is calculated as *τ* ≈ *d*/*v* ≈ 1 s, which is close to the observed value, 0.1 s. Transport by convective flow may thus account for the delay. Considering the above three processes, the second and third candidates, i.e., mass transport by diffusion and that by convective flow, are important for the droplet deformation following the velocity change by the time delay of approximately 0.1 s.

**Figure 4:**
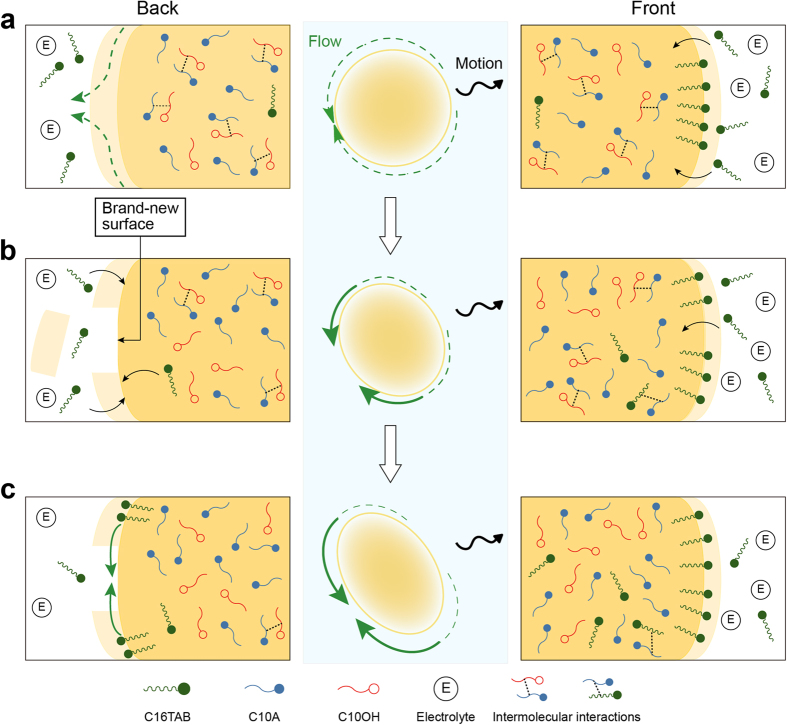
Proposed mechanism for deformable self-propelled oil droplets. (**a**) Self-propelled motion of oil droplet. (**b**) Self-propelled motion of oil droplet exhibiting slight deformation owing to the generation of a brand-new surface at the back induced by the fast flow around the droplet. (**c**) Self-propelled motion of oil droplet exhibiting deformation due to the precipitous gradient fields of interfacial tension at the back.

From the viewpoint of diffusion and convective flow at the molecular level, we can propose a mechanism for the deformable self-propelled oil droplets (Fig. 4). The self-propelled motion of oil droplets can be interpreted as following three stages. First, surfactant molecules begin to adsorb onto the droplet surfaces, and heterogeneity of droplet surfaces is induced by non-uniform surfactant concentrations, as well as by thermal fluctuations. This causes an imbalance in the interfacial tension between sites with adsorbed surfactant molecules and bare sites on the droplet surface. Next, the flow at the oil droplet surface due to the imbalance of the interfacial tension is maintained by Marangoni instability, indicating symmetry-breaking^8^. Marangoni flow and subsequent mass transfer are likely caused by intermolecular interactions between the surfactant and oil molecules. In the third stage, the momentum between inside and outside of the droplet is exchanged through the Marangoni flow, and the droplet itself is driven in a certain direction. The more surfactant the moving droplet takes on, the more the droplet continues to move because of the flux balance between the oil droplet and the bulk. However, in the current system, C16TAB molecules can rapidly adsorb onto the oil‒water interface of the self-propelled droplets because of the reduction of cationic charge repulsion between surfactant molecules in the presence of electrolytes (HCl, NaCl, and so on) (Fig. 4a). Thus, faster flow is generated at the oil‒water interface (Fig. 4b). Actually, we confirmed from the analysis of outer flow fields using hydrophilic fluorescent microbeads that the flow at the back was stronger than that at the front in the self-propelled droplets exhibiting deformation. Such fast flow suggests a rupture of the droplet membrane at the back of the self-propelled droplets. Because the brand-new surface subsequently expands sideways owing to local peeling-off of some amount of the membrane, precipitous gradient fields of interfacial tension are locally formed only in this area. The rapid adsorption of C16TAB molecules at the front keeps the interfacial tension lower at the front (Fig. 4c). This rupture of the droplet membrane plays a key role in the deformable self-propelled droplets based on the formation of interfacial tension gradient fields and subsequent flow fields. Furthermore, from the results of DSC measurements, the mixture of C10A/C10OH (60/40 mol%) formed some harder phases in droplets owing to intermolecular interactions. However, because of the presence of electrolytes, relatively large amounts of C16TAB are taken up by self-propelled oil droplets. These induce acetal production and simultaneously the formation of softer phases owing to the intermolecular interaction change from C10A‒C10OH to the four component mixture, suggesting that the phases within the self-propelled oil droplets spatiotemporally change between “hard” and “soft” states. Such dynamic behaviors induce the localization of convective rolls inside the droplet, leading to its deformation.

To recap, the mechanism suggested here is consistent with that suggested from the data of the time delay of approximately 0.1 s between velocity and deformation changes, i.e., mass transport by diffusion and convective flow. As for the deformable self-propelled droplets, we consider the following two aspects. First, the membrane formation at the interface decreases the interfacial tension and allows deformation of the droplet. Second, the localized interfacial tension gradient and intermolecular interactions among surfactant and oil molecules drive the droplet, which can cause the localization of convective rolls inside the droplet. This localization of the convective rolls can induce the deformation.

In summary, we have demonstrated self-propelled motion with deformation using micrometer-sized oil droplets composed of a fatty aldehyde and a fatty alcohol in a cationic surfactant solution containing HCl. There were two reasons for the unique dynamics of oil droplets: the formation of a precipitous gradient field of interfacial tension at the back of droplet surface and the localization of convective rolls inside the droplet caused by both the localized interfacial tension gradient and the spatiotemporal switching of intermolecular interactions among surfactant and oil molecules. From the viewpoint of molecular science, the properties of deformable self-propelled droplets, such as motion time and speed, can be controlled by combining surfactant and oil molecules in the presence of electrolytes. Such a strategy is highly effective for the application of droplets as carriers and probes using micrometer-sized soft matter. We thus consider that these findings are important for scientific frontiers such as developing deformable micro-swimmers and exploring the emergence of self-locomotion of oil droplet-type artificial cells as a way of understanding protocell locomotion^30^.

## Methods

### Materials

Commercially-available reagents and solvents were purchased from Tokyo Chemical Industry Co. (Tokyo, Japan) and Kanto Chemical Co. (Tokyo, Japan), respectively, and were used without further purification. Water was distilled and deionized before use with a MilliQ system from Millipore (MA, USA).

### Synthesis of 1-didecyloxyundecane (diC10A)

To evaluate the effect of C10A and C10OH on the dynamics of oil droplets, diC10A was synthesized according to Madsen’s report^31^ with some modification. C10A (1.70 g, 10 mmol), C10OH (3.81 g, 24 mmol), *p*-toluenesulfonic acid monohydrate (0.38 g, 2 mmol), and activated 3A molecular sieves (2 g) were added to anhydrous hexane (10 mL) at 0 °C. The mixture was stirred for 24 h at room temperature before quenching with solid NaHCO_3_. The mixture was then filtered through celite, and the filtrate was dried under reduced pressure to afford a viscous colorless liquid. The liquid was dissolved in hexane (20 mL), rinsed once with 5% NaHCO_3_ aq (20 mL) and twice with water (20 mL), and then dried over anhydrous magnesium sulfate. The hexane was removed under reduced pressure and methanol (25 mL) was added to the residue resulting in biphase system. The lower layer was collected and the remaining methanol in the layer was evaporated for 12 h in vacuum to obtain diC10A as a colorless liquid at a yield of 45% (4.5 mmol, 2.13 g).

^1^H NMR (500 MHz, acetone-*d*_6_): δ 4.47 (1 H, t, *J* = 5.5 Hz), 3.48 (4 H, ddt, *J* = 78.5, 9.0, 6.5 Hz), 1.62−1.52 (6 H, m), 1.44–1.20 (44 H, m), 0.90 (9 H, t, *J* = 6.7 Hz).

### Optical microscopic observation of oil droplet dynamics

The observation specimen was prepared as follows. An emulsion of mixed C10A/C10OH was formed by agitating 200 μL of the C16TAB solution with a mixture of C10A/C10OH (10 μL). Immediately after mixing C10A and C10OH in the surfactant solution and encasing the emulsion sample in a thin glass-chamber (15 × 15 × 0.28 mm; Frame Seal Chamber, MJ Research Inc., Waltham), real-time observations were conducted at room temperature (23–25 °C) using a phase contrast microscope (IX71, Olympus, Japan) equipped with a charge coupled device camera (DP72, Olympus, Japan).

### Time course measurements of the reactions between C10A and C10OH

A mixture of C10A/C10OH (10 μL) was added to an aqueous solution in the presence of a 50 mM C16TAB solution (200 μL) at room temperature, lyophilized, and dissolved in CDCl_3_. The production of diC10A was analyzed by ^1^H NMR spectral measurements of the CDCl_3_ solution of C16TAB and products, and the percentage production of diC10A was calculated by integration of the ratio of the signals for acetal methine protons at δ 4.46 ppm to that for the signal for trimethylammonium from C16TAB protons at δ 3.47 ppm as an internal standard peak (see Supplementary Fig. 2).

## Additional Information

**How to cite this article**: Banno, T. *et al.* Deformable Self-Propelled Micro-Object Comprising Underwater Oil Droplets. *Sci. Rep.* **6**, 31292; doi: 10.1038/srep31292 (2016).

## Supplementary Material

Supplementary Information

Supplementary Information

Supplementary Movie 1

Supplementary Movie 2

## Figures and Tables

**Figure 1:**
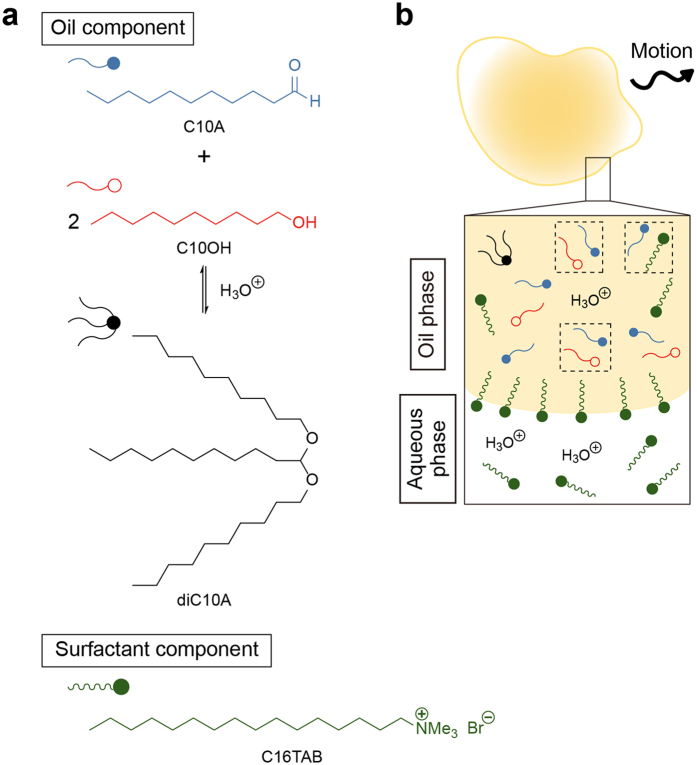
Concept of the creation of deformable self-propelled oil droplet system underwater. (**a**) Oil and surfactant components in the current system. diC10A is produced by dehydrocondensation of C10A and C10OH under acidic conditions. (**b**) Schematic representation of deformable self-propelled oil droplet. Self-propelled motion with deformation is induced by spatiotemporal heterogeneity at the surface and core of the oil droplet. Black dotted squares represent the attraction between surfactant and oil molecules.
