# Supplementary material for: Deformable Self-Propelled Micro-Object Comprising Underwater Oil Droplets
Source: Sci Rep. 2016 Aug 9;6:31292. doi: 10.1038/srep31292 (PMC4977503; doi:10.1038/srep31292)
Supplement: Supplementary Information [file srep31292-s1.pdf]

Supplementary Information

Deformable Self-Propelled Micro-Object Comprising Underwater Oil Droplets

Taisuke Banno[1], Arisa Asami[2], Naoko Ueno[2], Hiroyuki Kitahata[3], Yuki Koyano[3], Kouichi Asakura[1], and Taro Toyota[2, 4, *]

[1] Department of Applied Chemistry, Faculty of Science and Technology, Keio University, 3-14-1 Hiyoshi, Kohoku-ku, Yokohama 223-8522, Japan
[2] Department of Basic Science, Graduate School of Arts and Sciences, The University of Tokyo, 3-8-1 Komaba, Meguro-ku, Tokyo 153-8902, Japan
[3] Department of Physics, Graduate School of Science, Chiba University, 1-33 Yayoi-cho, Inage-ku, Chiba 263-8522, Japan
[4] Research Center for Complex Systems Biology, The University of Tokyo, 3-8-1 Komaba, Meguro-ku, Tokyo 153-8902, Japan.

*Correspondence to: Prof. Taro Toyota, Department of Basic Science, Graduate School of Arts and Sciences, The University of Tokyo, 3-8-1 Komaba, Meguro-ku, Tokyo 153-8902, Japan; Fax: +81-3-5465-7634; Tel: +81-3-5465-7634; E-mail: cttoyota@mail.ecc.u-tokyo.ac.jp

Supplementary Information Index

Method of fitting of oil droplet shape and analysis on the elliptic deformation … p.2

Figures … p.6

Tables … p.8

Video clip description …p.10

Method of fitting of oil droplet shape and analysis on the elliptic deformation

We obtained a movie of the droplet and made a binary image of the droplet by setting a threshold. In this process, we extracted only one droplet by deleting the images of other droplets and small objects such as aggregates of surfactants.

Then, we calculated the coordinates of the center of mass of the droplet, $(\langle x\rangle, \langle y\rangle)$ as

$$\langle x\rangle = \frac{1}{N}\sum_{i=1}^{N} x_i, \tag{1}$$

$$\langle y\rangle = \frac{1}{N}\sum_{i=1}^{N} y_i, \tag{2}$$

and the second-order moments of the droplet as

$$M_{XX} = \langle (x-\langle x\rangle)^2\rangle = \frac{1}{N}\sum_{i=1}^{N}(x_i-\langle x\rangle)^2\ , \tag{3}$$

$$M_{XY} = \langle (x-\langle x\rangle)(y-\langle y\rangle)\rangle = \frac{1}{N}\sum_{i=1}^{N}(x_i-\langle x\rangle)(y_i-\langle y\rangle)\ , \tag{4}$$

$$M_{YY} = \langle (y-\langle y\rangle)^2\rangle = \frac{1}{N}\sum_{i=1}^{N}(y_i-\langle y\rangle)^2\ , \tag{5}$$

where (x_i, y_i) is the position of the i-th pixel of N pixels that comprise the droplet.

Using these values, we considered a symmetric traceless matrix M as

$$M = \begin{pmatrix} (M_{XX}-M_{YY})/2 & M_{XY} \\ M_{XY} & (M_{YY}-M_{XX})/2 \end{pmatrix}. \tag{6}$$

From the matrix M, we defined the amplitude of deformation, s, and the angle of the elongation, θ, as

$$s = \sqrt{\left(\frac{M_{XX}-M_{YY}}{2}\right)^2 + {M_{XY}}^2}, \tag{7}$$

$$\theta = \frac{1}{2}\tan^{-1}\frac{2M_{XY}}{M_{XX}-M_{YY}}, \tag{8}$$

where $0 \le \theta < \pi$. Then, the matrix M was rewritten as

$$M = s\begin{pmatrix} \cos 2\theta & \sin 2\theta \\ \sin 2\theta & -\cos 2\theta \end{pmatrix}. \tag{9}$$

It is noted that the eigenvalues of M are $\pm s$ and that s is invariant with rotation around the center of mass, $(\langle x\rangle, \langle y\rangle)$. θ is regarded as the major axis direction in an elliptic approximation.

The representations of s and θ were confirmed as follows. First, we considered an ellipse whose major and minor axes were a and b, respectively. We considered the situation in which the center of the ellipse was located at the origin and the major axis was directed at ϕ. The moments were calculated as

$$M_{XX} = \langle x^2\rangle$$

$$= \frac{1}{A}\left[2\int_{-a}^{a}\int_{0}^{b\sqrt{1-x^2/a^2}}(x\cos\phi - y\sin\phi)^2 dydx\right]$$

$$= \frac{1}{\pi ab}\frac{\pi ab}{4}(a^2\cos^2\phi + b^2\sin^2\phi)$$

$$= \frac{a^2\cos^2\phi + b^2\sin^2\phi}{4}, \quad (10)$$

$$M_{XY} = \langle xy\rangle$$

$$= \frac{1}{A}\left[2\int_{-a}^{a}\int_{0}^{b\sqrt{1-x^2/a^2}}(x\cos\phi - y\sin\phi)(x\sin\phi + y\cos\phi) dydx\right]$$

$$= \frac{1}{\pi ab}\frac{\pi ab(a^2-b^2)\sin\phi\cos\phi}{4}$$

$$= \frac{(a^2-b^2)\sin\phi\cos\phi}{4}, \quad (11)$$

$$M_{YY} = \langle y^2\rangle$$

$$= \frac{1}{A}\left[2\int_{-a}^{a}\int_{0}^{b\sqrt{1-x^2/a^2}}(x\sin\phi + y\cos\phi)^2 dydx\right]$$

$$= \frac{1}{\pi ab}\frac{\pi ab}{4}(a^2\cos^2\phi + b^2\sin^2\phi)$$

$$= \frac{a^2\sin^2\phi + b^2\cos^2\phi}{4}, \quad (12)$$

where A is the area of the ellipse, πab, and the matrix M is explicitly written as

$$M = \frac{a^2 - b^2}{8}\begin{pmatrix} \cos 2\phi & \sin 2\phi \\ \sin 2\phi & -\cos 2\phi \end{pmatrix}. \tag{13}$$

Therefore, we confirmed that the angle, θ, corresponds to the angle of the major axis, ϕ, when the droplet shape is fitted as an ellipse. The amplitude, s, corresponds to $(a^2 - b^2)/8$, which is an increasing function of the difference between the major and minor axes. It is noted that $s = 0$ means that the shape is fitted as a circle rather than an ellipse.

Secondly, we considered a shape described as

$$R(\phi) = R_0(1 + \varepsilon \cos 2(\phi - \phi_0)), \tag{14}$$

in polar coordinates. The area A was calculated as

$$A = \int_0^{2\pi} \int_0^{R(1+\varepsilon\cos 2(\phi-\phi_0))} r dr d\phi = \pi R^2 \left(1 + \frac{\varepsilon^2}{2}\right), \tag{15}$$

and the moments were calculated as

$$\begin{aligned} M_{XX} &= \langle x^2 \rangle \\ &= \frac{1}{A}\left[\int_0^{2\pi} \int_0^{R(1+\varepsilon\cos 2(\phi-\phi_0))} (r\cos\phi)^2 r dr d\phi\right] \\ &= \frac{1}{A}\frac{\pi R^4}{4}\left(1 + 3\varepsilon^2 + \frac{3}{8}\varepsilon^4\right) + \frac{1}{A}\frac{\pi R^4}{2}\left(\varepsilon + \frac{3}{4}\varepsilon^3\right)\cos^2\phi_0, \end{aligned} \tag{16}$$

$$\begin{aligned} M_{XY} &= \langle xy \rangle \\ &= \frac{1}{A}\left[\int_0^{2\pi} \int_0^{R(1+\varepsilon\cos 2(\phi-\phi_0))} r^2 \sin\phi \cos\phi \, r dr d\phi\right] \\ &= \frac{1}{A}\frac{\pi R^4}{2}\left(\varepsilon + \frac{3}{4}\varepsilon^3\right)\sin\phi_0 \cos\phi_0, \end{aligned} \tag{17}$$

$$\begin{aligned} M_{YY} &= \langle y^2 \rangle \\ &= \frac{1}{A}\left[\int_0^{2\pi} \int_0^{R(1+\varepsilon\cos 2(\phi-\phi_0))} (r\sin\phi)^2 r dr d\phi\right] \\ &= \frac{1}{A}\frac{\pi R^4}{4}\left(1 + 3\varepsilon^2 + \frac{3}{8}\varepsilon^4\right) - \frac{1}{A}\frac{\pi R^4}{2}\left(\varepsilon + \frac{3}{4}\varepsilon^3\right)\cos^2\phi_0, \end{aligned} \tag{18}$$

and thus the matrix M is explicitly written as

$$M = \frac{\varepsilon R^2}{2+\varepsilon^2}\left(1 + \frac{3}{4}\varepsilon^2\right)\begin{pmatrix}\cos 2\phi_0 & \sin 2\phi_0 \\ \sin 2\phi_0 & -\cos 2\phi_0\end{pmatrix}. \quad (19)$$

In this case, the angle, θ, also corresponds to the angle, ϕ_0. The amplitude, s, is an increasing function of ε, and $s = 0$ when $\varepsilon = 0$. When ε is infinitesimally small, s is approximated as $s = \varepsilon R^2/2$.

To compare the self-propelled motion of deformable droplets with that of non-deformable ones, we investigated the relationship between the s and velocity using deformable C10A/C10OH and non-deformable C10A droplets. As a result, the s values of former were clearly higher than those of latter (see Fig. S1).

Figures

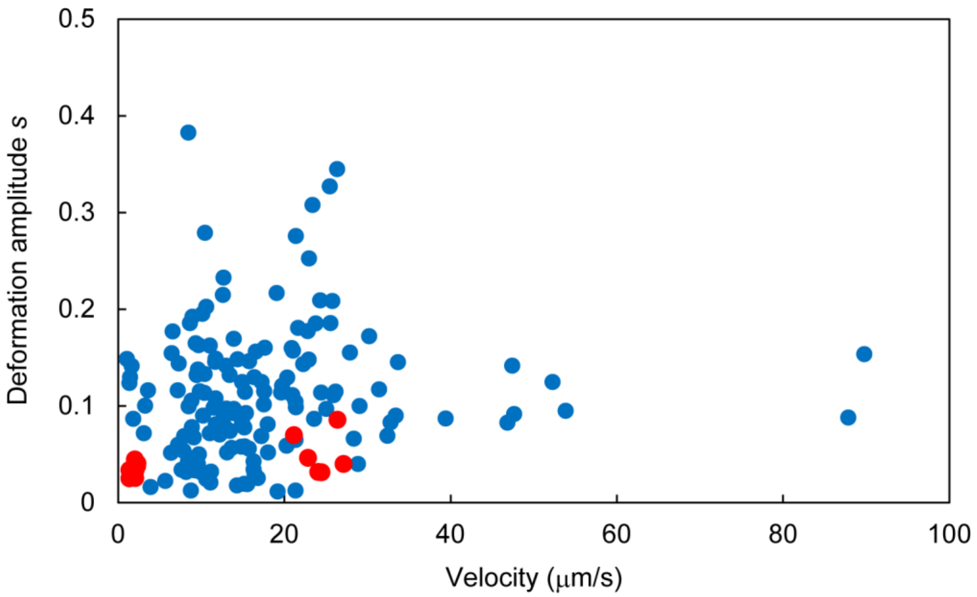

Figure S1. Relationship between the *s* and velocity using deformable C10A/C10OH and non-deformable C10A droplets with diameters of 10–150 μm. The analyzed emulsion samples were prepared by dispersing 10 μL of mixed C10A/C10OH (blue solid circles) or C10A (red solid circles) into 50 mM C16TAB containing 0.01 M HCl (200 μL) at room temperature (23–25 °C). Each plot is the average for 0.7 s.

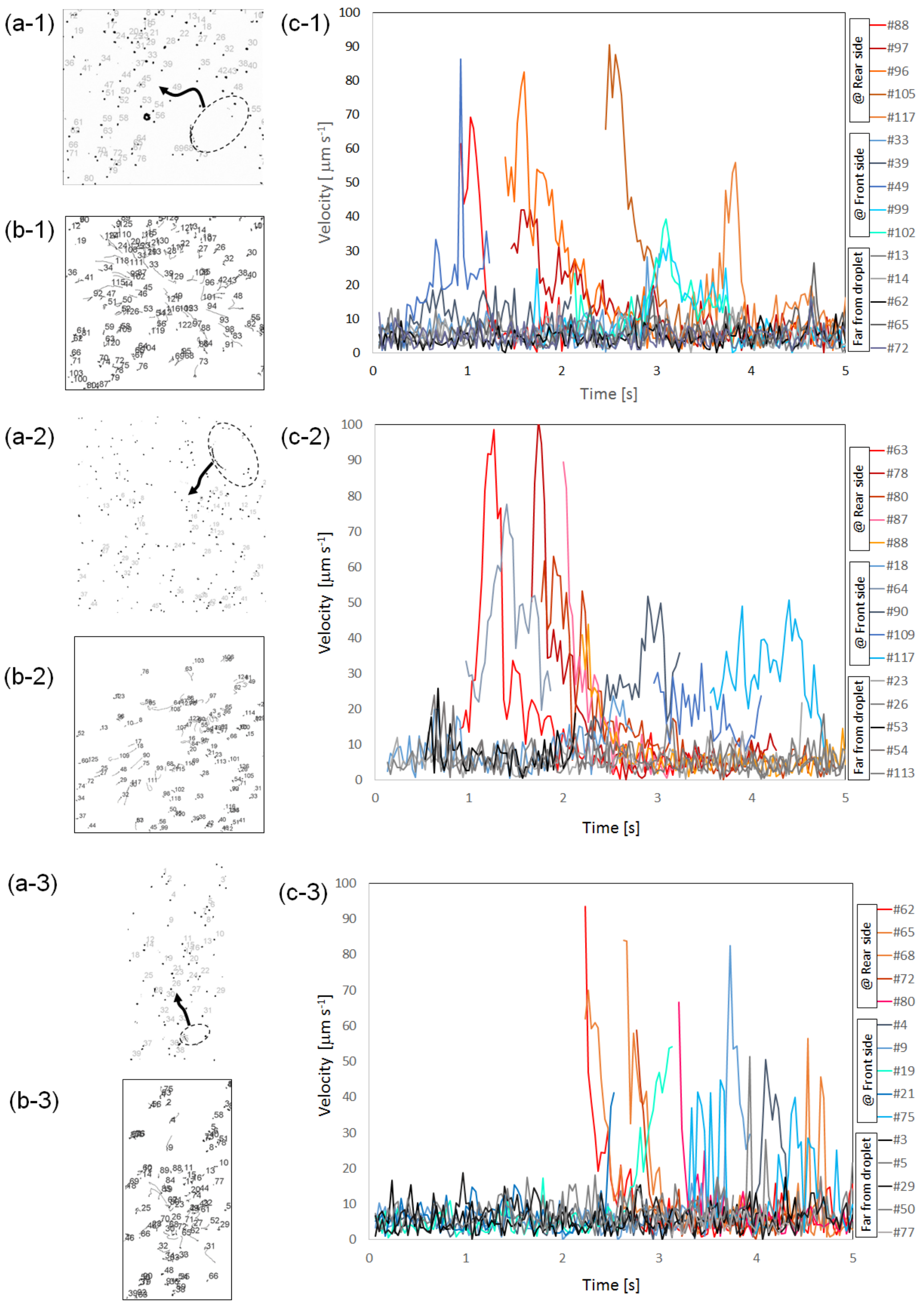

Figure S2. Analysis of flow around the three deformed self-propelled oil droplets using water-dispersed fluorescent microspheres (diameter = 1 μm). (a) Initial position of the oil droplets and the fluorescent microspheres (indicated by the numbers). (b) Trajectories of

fluorescent microspheres for 5 s. (c) Time series of the flow speed of each fluorescent microsphere in (b). The dotted line and black arrow in (a) represent the rim of the deformed self-propelled oil droplet in the initial position (t = 0 s) and the direction of self-propelled motion, respectively (see Movie S2 for (a-1)).

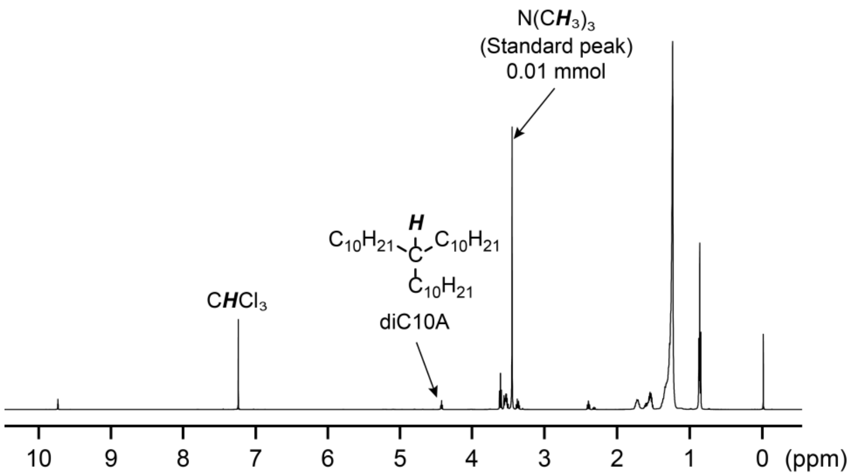

Figure S3. ^{1}H NMR spectrum ($CDCl_3$) of the reaction products of C10A (0.03 mmol) and C10OH (0.02 mmol) in 50 mM C16TAB containing 0.001 M HCl (200 μL) at room temperature after 60 min.

Tables

Table S1 Effect of C16TAB concentration on the dynamics of oil droplets composed of C10A/C10OH (60/40 mol%) in 0.01 M HCl at room temperature.

Entry	C16TAB (mM)	Motion time (min)	Deformation	Duration of self-propelled motion with deformation (min)*
1	5	n. o.	n. o.	n. o.
2	10	29	✓	4
3	20	>60	✓	5
4	30	11	✓	2
5	40	26	✓	26
6	50	>60	✓	>60

*From start of the observation. n. o. = not observed.

Table S2 Effect of the C10A to C10OH molar ratio on the dynamics of self-propelled oil droplets in 50 mM C16TAB solution containing 0.01 M HCl at room temperature.

Entry	Molar ratio (mol%)		Motion time (min)	Morphology of oil droplets
	C10A	C10OH		
1	100	0	19	Spherical
2	80	20	26	Spherical
3	60	40	>60	Deformed shape
4	40	60	3	Deformed shape
5	20	80	30	Deformed shape
6	0	100	n. o.	Spherical

n. o. = not observed.

Table S3 Effect of HCl concentration on the dynamics of oil droplets composed of C10A/C10OH (60/40 mol%) in 50 mM C16TAB solution at room temperature.

Entry	Dispersion	Motion time (min)	Deformation	diC10A (%)*
1	Water	25	n. o.	0.3
2	0.001 M HCl	29	n. o.	9
3	0.01 M HCl	>60	✓	3
4	0.1 M HCl	12	✓	4

*After 60 min. The maximum production was 33 mol%. n. o. = not observed.

3% and 4% of diC10A was produced after 60 min of reaction in 0.01 M and 0.1 M HCl, respectively, whereas only 0.3% of diC10A was generated in water. For 0.001 M HCl, the production of diC10A was 9%. This may have been due to the slower hydrolysis of diC10A in 0.001 M HCl than in 0.01 M or 0.1 M HCl.

Table S4 Effect of additional diC10A concentration on the dynamics of oil droplets composed of C10A/C10OH (60/40 mol%) in 50 mM C16TAB solution containing 0.01 M HCl at room temperature.

Entry	Dispersion	diC10A (mol%)*	Motion time (min)	Deformation
1	0.01 M HCl	0	>60	✓
2	0.01 M HCl	1	35	✓
3	0.01 M HCl	3	33	n. o.
4	0.01 M HCl	5	10	n. o.
5	0.01 M HCl	10	n. o.	n. o.

* Relative to C10A/C10OH oil components. n. o. = not observed.

Table S5 Effect of electrolyte concentration on the dynamics of oil droplets composed of C10A/C10OH (60/40 mol%) in 50 mM C16TAB solution at room temperature.

Entry	Dispersion	Motion time (min)	Deformation
1	0.001 M NaCl	>60	✓
2	0.01 M NaCl	>60	✓
3	0.1 M NaCl	21	✓
4	0.01 M NaBr	45	✓
5	0.01 M $MgCl_2$	>60	✓
6	Water	25	n. o.

n. o. = not observed.

Video clip description

Two video clips are attached to show the deformation of self-propelled oil droplets composed of C10A and C10OH in a solution of C16TAB (50 mM) containing 0.01 M HCl.

Movie S1: Typical deformed self-propelled oil droplets in C16TAB solution containing HCl.

Movie S2: Visualization of the flow fields around a self-propelled oil droplet exhibiting deformation using water-dispersed fluorescent microspheres (diameter = 1 μm).
